# Importance of on-farm research for validating process-based models of climate-smart agriculture

**DOI:** 10.1186/s13021-024-00260-6

**Published:** 2024-05-29

**Authors:** Elizabeth Ellis, Keith Paustian

**Affiliations:** 1 Department of Soil and Crop Sciences, Colorado State University, Fort Collins, CO USA; 2 Natural Resource Ecology Laboratory, Colorado State University, Fort Collins, CO USA

## Abstract

Climate-smart agriculture can be used to build soil carbon stocks, decrease agricultural greenhouse gas (GHG) emissions, and increase agronomic resilience to climate pressures. The US recently declared its commitment to include the agricultural sector as part of an overall climate-mitigation strategy, and with this comes the need for robust, scientifically valid tools for agricultural GHG flux measurements and modeling. If agriculture is to contribute significantly to climate mitigation, practice adoption should be incentivized on as much land area as possible and mitigation benefits should be accurately quantified. Process-based models are parameterized on data from a limited number of long-term agricultural experiments, which may not fully reflect outcomes on working farms. Space-for-time substitution, paired studies, and long-term monitoring of SOC stocks and GHG emissions on commercial farms using a variety of climate-smart management systems can validate findings from long-term agricultural experiments and provide data for process-based model improvements. Here, we describe a project that worked collaboratively with commercial producers in the Midwest to directly measure and model the soil organic carbon (SOC) stocks of their farms at the field scale. We describe this study, and several unexpected challenges encountered, to facilitate further on-farm data collection and the creation of a secure database of on-farm SOC stock measurements.

## Introduction

While agriculture is a major source of GHG emissions, agriculture can also contribute to climate mitigation through avoided emissions and carbon sequestration by following conservation management principles [1, 6, 7, 44]. The United States agricultural sector contributes approximately 10% of total US greenhouse gas (GHG) emissions. While only a small proportion of total agricultural emissions (< 1%) currently come from the conversion of grassland and forest to croplands [32], creation of new agricultural land and the release of soil carbon (C) as CO_2_ was a significant historical source of emissions to the atmosphere [60]. Thus, a significant C ‘deficit’ remains in most US cropland soils due to historical land use change, generated through lower rates of plant C inputs to soil compared to native ecosystems, along with decades of intensive tillage and extended bare fallow periods [24, 55]. Restoring SOC lost from cropland soils can contribute to climate mitigation, reduce GHG emissions associated with agricultural activities, and regenerate degraded land.

Practices that rebuild SOC stocks have the added benefits of restoring soil fertility, reducing emissions associated with field activities, and increasing agroecosystem resilience to climate stressors, such as drought, flooding, and pest pressures [6, 7, 25]. Thus, these practices fall under the umbrella of “climate-smart agriculture,” indicating they are tools to both mitigate atmospheric C and adapt to the pressures of climate change [12, 44]. Soil carbon stocks can be replenished over time by managing the balance between photosynthesis (C gain) and biological decomposition (C loss). During decomposition, the majority of plant residue C is lost as CO_2_ to the atmosphere while a portion is stabilized in SOM as soil organic matter carbon (SOM-C or SOC). If the amount of C added to the SOC pool exceeds the amount lost as CO_2_ during decomposition, cropland soils act as a net sink for atmospheric C. This can occur with changes in management practices that induce greater plant C inputs and/or reduce SOM decomposition rates, although there is a finite capacity or ‘saturation’ limit to the attainable size of C stocks in agricultural soils [63] There are several climate-smart practices that can positively impact the soil C balance, such as no-till, cover crops, diversified crop rotations, integrating animals into crop systems, or a combination of multiple synergistic practices [2, 6, 21, 43, 44].

With recent national and international commitments to agriculture as a climate mitigation strategy [33, 68], there is demand for reliable methods to measure and estimate changes in SOC stocks and GHG fluxes under agricultural management change. Ecosystem process-based models are increasingly used to estimate SOC stocks and GHG fluxes at the field and regional level. The applicability of models is dependent on the degree to which they are parameterized, calibrated, and validated to reflect the environmental and management characteristics of the unique land use settings and soil characteristics encountered across the country [37]. Thus, to accurately quantify or estimate the climate mitigation potential of climate-smart practices, it’s important that measurements and models reflect the variety of soil types, topography, and management systems encountered on cropland across the country. Here, we make the case for increased sampling and activity data collection on commercial farms to test conclusions from long-term field experiments (LTEs), improve process-based models, and support the successful adoption of climate-smart practices at scale. We describe challenges in process-based modeling associated with data availability, summarize recent studies of SOC stock change in commercial farm settings, and propose a method for integrating on-farm measurements and activity data to improve model simulations.

## The US is committed to agriculture as a climate-mitigation strategy, but available quantification tools need improvement

The US agricultural sector declared its commitment to agriculture as a climate-mitigation strategy with the announcement of USDA’s Partnerships for Climate Smart Commodities project [45] and the significant allocation of Inflation Reduction Act funds for currently oversubscribed agriculture conservation programs, like CSP and EQIP [23]. Collectively, these two initiatives appropriated $22.6 billion towards the creation of markets for climate-smart commodities and to support agricultural producers in the adoption of climate-smart practices to both mitigate and adapt to the pressures of climate change. If climate-smart practices are adopted on all possible US agricultural land, it is estimated that 0.17 Gt CO_2_e yr^−1^ could be sequestered over the following 20 years [61]. By the end of the century, a total mitigation of 110 Gt CO_2_e might be achieved in the US through a range of nature-based climate mitigation practices on managed lands, including C sequestration in soils and biomass and bioenergy with carbon capture and storage [52]. However, catalyzing wide-spread management changes on millions of farms and ranches across the US continues to be a major challenge [53, 58]. Technical assistance, social change, and economic incentives are needed across scales and sectors to spread the use of climate-smart practices [28, 48]). One such incentive is the creation of markets for agriculturally-generated carbon credits that pay producers for C stored in their soil and/or reduced GHG emissions [39, 40], an otherwise unrewarded ecosystem service [50].

A common goal of private carbon credit markets and other climate-smart initiatives, such as ‘low-carbon’ supply chains, is to improve methods for monitoring, reporting, and verification (MRV) of the GHG emission reductions or C sequestration benefits of climate-smart practices. Most MRV protocols require a combination of direct soil measurements, modeling, and/or remote sensing, but there are no industry or governmental standards to ensure the comparability of offsets generated through different protocols [39]. With current methods and technologies, laboratory measurement of soil carbon is too costly and time-intensive to be used on all desired fields and with the large sample sizes needed to detect small changes in carbon stocks [8, 62]. Thus, a combination of direct measurement and process-based biogeochemical modeling represents the “gold standard” for voluntary carbon-market MRV protocols [14, 43, 59].

Nearly all process-based model development processes are carried out using field activity data and SOC and/or GHG flux measurements from LTEs with controlled and repeated management. Although LTEs are essential for basic agricultural research and model parameterization, they are limited in their extent and representation of soil, climate, topography, field size, and management combinations, and therefore do not include the full range of activities, outcomes, and production challenges encountered on real commercial farms [5, 17, 18]. There is need for greater collaboration between researchers, agricultural producers, and various entities developing MRV protocols to improve process based models using on-farm measurements and activity data [8, 40].

## Model-based simulations of SOC stocks could be improved with further on-farm measurements.

Simulation of ecosystem processes for estimation of changes in SOC stock and GHG emissions are, and will continue to be, an essential aspect of climate-smart agriculture MRV protocols. Process-based biogeochemical models, such as DayCent [41], DNDC [19], and RothC [13], rely on measurement data to improve simulations of agroecosystem processes occurring above and belowground and their subsequent impacts on biogeochemical stocks and fluxes, including SOC stocks and GHG emissions. SOM models and modeling approaches incorporate new experimental data to reflect the latest understanding of SOM dynamics, such as saturation kinetics, permanence, priming, measurable SOM fractions, subsoil SOM, and specific microbial processes [3, 10, 11, 57, 69]. Despite improvements in the ability of process-based models to simulate observed soil C dynamics, uncertainties remain high when estimating SOC stock change at the field and regional scale. For example, uncertainties for simulating past SOC stock change in the Century model were as high as ± 118% and ± 739% at subregional and site scales, respectively [38]. Bayesian model analysis frameworks and intermodal comparisons have proven useful for reducing uncertainty [22, 64], but the limited amount of measured data for model calibration and validation remain the largest bottle-neck for further reducing uncertainty in quantifying SOC stock changes [4, 10, 14, 22, 47].

New modeling techniques incorporating artificial intelligence and multi-model ensembles may improve simulation accuracy and uncertainty estimations if the necessary data are available. For example, machine learning (ML) algorithms have the potential to incorporate large quantities of data to model ecological processes with superior performance to traditional process-based models [4]. Furthermore, multi-model ensembles that incorporate the strengths of several models simultaneously are shown to improve C flux simulations and are currently being pursued by collaborative groups throughout the process-based modeling community [51, 56, 64]. Even more so than traditional process-based models, the performance of ML algorithms and multi-model ensembles is strongly dependent on the quantity and quality of measurement data used for training, calibration, and validation.

Most data available for model development are generated at LTEs and LTE networks representing a narrow range of agricultural regions, crop production systems, climate-smart practices, topographical settings, and soil types [49]. While LTE data are essential for generating high-control, low-variability, time series data for model parameterization, applicability of experimental results to the broader range of commercial farm settings is uncertain. When modeling outcomes on commercial farms, accurately simulating biomass input (crop yields), impacts of historical land use, and baseline SOC stocks is important [31]. Furthermore, available data must represent the full range of pedo-climatic and landscape-level variables (including topography and soil erodibility) that affect SOC baseline stocks, SOC accrual, and SOC stability in commercial farms settings [9, 65, 69]. In the following sections, we provide examples of on-farm studies that help to address the lack of data from commercial farm settings. We then outline how on-farm direct and remotely-sensed measurements could be incorporated into a secure data platform to facilitate further model improvements.

## Observational experimental techniques popular in other subfields of ecology can be used to generate on-farm measurement data.

To effectively use process-based models and decision support tools to quantify SOC stock change and GHG emissions on commercial farms, we need more measured soil data from real farms using a variety of management systems to supplement LTE data. On-farm monitoring data (i.e., measurements across multiple time points, ideally including baseline measurements) are essential to improve model calibration and validation, particularly as process-based model results are increasingly used in voluntary C market MRV protocols.

While LTEs enable us to quantify the impacts of specific management variables in comparison to a no-treatment control, the application of experimental results to real-world agronomic conditions has limitations [17, 18]. First, the agricultural inputs of LTEs (such as biocides, fertilizers, and organic amendments) are often held consistent from year to year, while commercial farms vary their management based on input prices, weather, success of previous years management, and personal agronomic goals [20]. Further, LTEs prioritize management consistency over maximizing yields, so yields of LTE plots often do not reflect yields on nearby commercial farms [26]. This discrepancy may impact the biomass input calibrations in process-based models. Perhaps most importantly, there are few existing LTEs that measure the impacts of multiple synergistic management practices applied to the same field. For example, a Midwest farmer dedicated to soil regeneration may use no-till in combination with winter cover crops, manure injection, diversified rotations, and reduced synthetic N inputs. LTEs are not designed to fully reflect the real-world complexity of annual crop production under various economic, social, and ecological pressures.

Establishing new LTEs that reflect region-specific climate-smart management changes at the systems-level (i.e., multiple practices used simultaneously) would allow us to understand the synergistic outcomes of complex management systems [43], but in the absence of such experiments in the short term, we can adopt observational experiment techniques popular in other subfields of ecology, such as space-for-time substitution and paired field studies [46]. For example, space-for-time substitution experiments can be used to generate chronosequences of SOC stocks and/or GHG flux measurements from adjacent farms using contrasting management systems [26, 29, 46]. Additionally, recent studies have worked collaboratively with agricultural producers across select regions to sample commercial fields representing a broader range of climate-smart practices than are currently established in LTEs or to compare LTE results to on-farm realities [5, 17, 18, 34, 54, 66]. Table 1 summarizes several studies using one of the following approaches to generate and evaluate on-farm data: (1) space-for-time substitution, (2) paired fields, (3) comparison between commercial farms and LTEs, or (4) diversity of commercial farm management across a region.

**Table 1:** Description of recent studies employing various observational methods for on-farm data collection

	Type of study or purpose	Management system	Field selection method	Number of farms/fields	Variables of interest	Activity data collection methods
[5]	Paired on-farm study; Regional variability in practice use and soil, topography, climate variables; Comparison to LTE	No-till vs. conventional tillage; Comparison to undisturbed control; Range of cropping systems	Adjacent pairs only; Duration of no-till management, crops produced, and soil type varied widely across study pairs	11 MLRAs, one farm pair per MLRA; Compiled data from 16 published, paired LTEs for comparison	Whole profile (0–60 cm) SOC stocks, BD, C/N	Report years of current tillage practice and N fertilization rate; Method of data collection unknown
[17]	Paired on-farm study; Outcomes on commercial farms under a range of management; Comparison to native ecosystem SOC stocks; Comparison to LTE measurements	Conservation tillage, no-till, cropping intensification	Sites with long-term conservation practice use (20–30 years); Neighboring LTEs; Each agricultural site paired to a natural vegetation site	Nine paired sites (agriculture vs. natural vegetation); Four LTEs and five commercial farms	SOC stock; SOC recovery (to native ecosystem)	Report years of current and historic tillage practices, biomass input, and crop rotation; Method of data collection unknown
[18]	Comparison of commercial farms to LTEs; Outcomes on commercial farms under a range of management systems (not represented in LTEs)	Broad range of cropping systems across select region of Switzerland	Randomly selected from regional farm directory, randomly selected one field per farm	120 farms, one composite sample per farm	Change in topsoil (0–20 cm) SOC stock; SOC:clay	Pulled from records (record keeping mandatory in Switzerland to participate in subsidy programs); interviews with selected producers to gap fill and confirm
[29]	Chronosequence of land use change	Transitioning cropland to management-intensive grazing	Similar row crop management and tillage histories	Three farms representing a seven-year period of land use change	SOC stocks; CEC; root mass; δ^13^C	Report tillage history, forage crops seeded, cattle stocking rates, and rotation frequency; Method of data collection unknown
[34]	Paired on-farm study of grazing systems	Adaptive multi-paddock (AMP) grazing vs. conventional grazing	Survey to identify AMP grazers in the region, selected 25 for in person visits, identified conventional grazing neighbor, selected 5 best pairs based on soil type, management history, etc	Ten farms (five pairs); sampled two catenas per farm	SOC and N stocks; root C; SOM fractions	Surveys and site visits/interviews
[54]	Outcomes on commercial farms under a range of management systems (not represented in LTEs)	Dryland cropping system intensification	Producers identified through “snowball” sampling; sample represents a range of mangement conditions, soil types, PET rates, and crop rotations	96 fields (54 commercial farms, 42 LTEs, 6 CRP sites)	SOC stocks, microbial activity, aggregation	Five years of activity data collected direct from producer or LTE manager

Of course, the greatest limitation of paired farm comparisons and chronosequence designs is that they are *not* randomized, controlled experiments and thus, are associated with a high degree of variability, unpredictability, and study design challenges. Common challenges noted in the studies presented in Table 1 include accounting for spatial variability in SOC stocks at the field-level, identifying producers willing to allow access to their farms, obtaining reliable and complete activity data, and site selection to control for variability between sites. On-farm studies rely on the implicit assumption that differences between paired-fields or categorically grouped farms are attributable primarily to management system differences, and that the variables of interest (i.e., SOC stocks) were similar before the management change occurred, but this assumption is difficult to verify. Careful site selection can be used to ensure sampled fields have similar soil types, topographic position, and long-term management histories, but some uncertainty about the influence of non-management induced differences in soil carbon stocks often remains.

While on-farm analyses are essential for understanding the impact of management change as it is applied at the commercial farm scale, evaluating more than one management practice simultaneously, along with environmental co-variates that also impact SOC stocks, makes it difficult to determine which practice (i.e., cover cropping, tillage regime, manure additions, or fertilizer reduction) is driving the results. Thus, there is still great value in controlled experiments that manipulate one management variable at a time. On-farm measurement data should be used in combination with measurements from LTEs to evaluate the extent to which LTEs reflect on-farm outcomes and whether process-based models are biased to LTE measurements.

In the following section, we describe our personal experience designing and executing an on-farm study of SOC stock differences between conventional and climate-smart farms across a portion of the Upper Corn Belt region (Iowa and Southern Minnesota). In addition to the studies cited in Table 1, we hope our experience can serve as a case study of the benefits and challenges of on-farm research and provide a road map for further on-farm data collection efforts.

## On-farm SOC stock measurement and activity data collection is challenging, yet necessary.

To help address the paucity of SOC stock data from commercial farms, we conducted on-farm sampling and collected detailed management activity data from 22 farms across Iowa and Southern Minnesota. We describe our study design and activity data acquisition efforts, along with unexpected challenges encountered, to facilitate future on-farm sampling and activity data collection. A more comprehensive discussion of the measured data results and model simulations is currently in preparation and will be published in future papers.

Spatial variability in management systems allowed us to design a paired, space-for-time substitution experiment, where we identified neighboring producers using different management practices on the same soil type for “across-the-fence” comparisons of the soil C and N stocks and agronomic impacts of different management systems with a range of years since practice adoption. Despite a recent relative increase in cover crop adoption across the Midwest [70], there is still a high degree of variability in management systems across the region and between neighboring farms. Cover crops are used on about 4.2% of acres and no-till on 30% of acres in Iowa [35], indicating that climate-smart management is still far from the norm. Given the heterogeneity in regional practice use, it was possible to identify neighboring producers using contrasting management systems for a paired comparison of outcomes.

After harvest in the fall of 2022, we sampled 22 paired corn and soybean farms throughout Iowa and Southern Minnesota using one of two management systems: (1) “climate-smart” (defined as greater than 8 years of no-till and at least 4 years of cover crop use, along with other best management practices), or (2) “conventional” (defined as annual or biannual tillage and winter bare fallow, which is typical of most row crop production systems in Iowa). All farms were planted in a corn and soybeans rotation, while some also raised livestock and used manure as an input. To reduce variability associated with crop-year, all fields selected for sampling were planted to soybeans in 2022. Rather than isolating one management practice as the study “treatment”, such as tillage or cover crops, the study design allows us to evaluate the impact of common management systems that use complementary practices simultaneously and vary management from year to year. For each of the 22 farms we obtained a series of soil cores for four depth increments (0–15, 15–30, 30–60, and 60–100 cm) to enable direct measurements of soil organic C and N and other soil properties (including bulk density, pH, textural class, inorganic C content, and microbial community composition). Our paired, across-the-fence approach used space-for-time substitution to measure and model the impact of management change, where the conventional farms represent soil conditions pre-management change. The climate-smart farms adopted conservation practices at different times, allowing us to generate a chronosequence of measurements representing different times since practice adoption (while accounting for management differences and regional variability in soil properties and climate). We buried ball markers at each sampling location to enable precise resampling at the same location at a future time [15]. Long-term monitoring of each farm will be used to establish a time series of measurements documenting the impact of long-term practice use (i.e., SOC change with 5 vs. 15 years of cover crop use). Additionally, we asked each farmer to voluntarily provide year-by-year management information (going back to 2013 or further, if available) needed to schedule events in the DayCent model and the COMET-Farm decision support tool (Table 2).

**Table 2:** Yearly management data needed to schedule events in the DayCent process-based model and the COMET-Farm GHG accounting tool, along with the number of producers who were able/willing to provide data for each requested data point in our on-farm study

Period data are needed for	Data category	Specific activity data needed to schedule model events	# of producers reporting ≥ 10 years activity data	# of producers reporting ≥ 5 years activity data
*Pre 1980 and 1980-2000s*	Historic management	• **Pre-1980s land use (grazing, cropland, upland vs. lowland)**	• 0/22	• 0/22
		• Pre 2000 enrollment in CRP (yes or no)	• 0/22	• 0/22
		• **1980–2000 management: irrigated vs. non-irrigated, crop type vs. grazing, tillage practices**	• 0/22	• 0/22
*Baseline (2000 to start of management change scenario)*	Crop planting and harvest	• **Cash crop type/rotation**	• 15/22	• 19/22
		• **Planting date**	• 10/22	• 19/22
		• **Harvest date**	• 8/22	• 19/22
		• **Yields**	• 10/22	• 19/22
		• Residue removal (% dry matter)	• 1/2^a^	• 2/2^a^
	Cover crops	• **Presence of cover crop**	• 11/11	• 11/11
		• Cover crop type or mixture	• 9/11	• 9/11
		• Seed population	• 6/11	• 8/11
		• Planting date	• 6/11	• 8/11
		• Planting equipment	• 7/11	• 8/11
		• Termination/harvest date	• 6/11	• 7/11
	Grazing practices	• **Start and end date**	• 1/2^a^	• 1/2^a^
		• **Rest period (days)**	• 0/2	• 0/2
		• **Daily utilization %**	• 0/2	• 0/2
	Tillage and other implement passes	• Date of each implement pass	• 8/22	• 11/22
		• **Tillage intensity (intensive, reduced, no-till)**	• 12/22	• 19/22
		• Other implement passes (roller crimping, mowing, herbicide application)	• 0/22	• 3/22
	Irrigation	• **Presence of irrigation**	NA	NA
		• Irrigation start and end date		
		• Irrigation amount (inches per application)		
		• Days between irrigation events		
	Manure application	• **Presence of manure**	• 0/5^a^	• 0/5^a^
		• Date applied	• 4/5	• 4/5
		• Manure type and form (solid, slurry)	• 3/5	• 3/5
		• Manure application rate	• 0/5	• 0/5
		• Moisture % (if known)	• 0/5	• 0/5
		• Total N and C/N ratio (if known)		
	Fertilization	• Date applied	• 2/22	• 9/22
		• Fertilizer type applied	• 12/22	• 15/22
		• Total N applied (kg/ha) per event	• 11/22	• 14/22
	Liming	• Liming date	• 2/5^a^	• 4/5^a^
		• Liming material and amount applied	• 0/5	• 2/5
	Burning	• **If burning occurred, did it occur before planting or after harvest?**	NA	NA

Bold text = properties that can be remotely-sensed given current technologies

^a^assumed only to be applicable to specific farms and years

It is important to note the tradeoffs and challenges associated with an on-farm study of this kind: First, it is challenging to attribute differences in measurements between farms to a particular soil characteristic or management practice used. Lack of experimental control leads to other challenges such as a higher degree of variability across a single field and between pseudo-replicates, making it difficult to quantify effect size. While we did our best to control for variations in soil type, topography, and land use history at the study design phase, there were often uncontrolled differences between the pairs that were not identified until after SOC stock and activity data were analyzed. For example, after receiving unexpectedly low SOC stock measurements from a regenerative farm field, we learned from the producer that that field was highly degraded and low yielding when he transitioned it to no-till 30 years ago. In this case, the conventional *vs* regenerative pair had quite different management and erosive histories, violating the assumption that the baseline SOC stocks were similar. This issue could have been avoided through more careful field selection, perhaps through in-depth farmer interviews or remote sensing of historic land use.

Connecting with willing farmer participants was an additional unforeseen challenge of our study and may prove difficult for future research aiming to collect on-farm data. We identified farms using “snowball” sampling techniques, where each producer who agreed to participate in the study recommended nearby producers who might be willing to participate as well. Connecting with farmer networks such as Practical Farmers of Iowa, attending local field days, and gaining connections through agronomic consultants were helpful strategies in the participant recruitment process. Identifying neighboring producers with the same soil type, topography, and management history was challenging when connections could only be provided to specific neighbors.

Reaching a level of trust with each participant (in most cases, by visiting their farms before data collection began) or gaining an introduction through a trusted organization or individual was a necessary element of the recruitment process. When asking participants to divulge detailed management information and to talk openly about their farming expertise, it was important that participants knew how the data collected would be used and kept secure. Institutional Review Board (IRB) approval was required to collect this information and was used to establish structures for data security and confidentiality.

Beyond data security concerns, our data collection experience brought to light other difficulties associated with collecting management data from producers for process-based modeling. Process-based modeling at the field level requires detailed yearly activity data (ideally going back 20 or more years) and knowledge of historic land use practices, which must be provided by the land managers or ascertained through remote sensing. For example, scheduling management events in the DayCent model and the COMET-Farm decision support tool requires data on tillage practices, fertilization rates, crop cultivars, organic matter addition, grazing events, among other field activities, including the dates of each management action. Even with substantial efforts to build relationships and trust, obtaining detailed activity data from the producers involved in our study was still difficult. Commonly unreported or incompletely reported data included historic management practices, planting/harvest dates, tillage dates, tillage implement/intensity, fertilizer application dates, and detailed liming practices. Only one producer was able to provide activity data for all ten years and all applicable management categories, and this producer owns an agronomic consulting business that helps producers collate their management data for carbon crediting programs, among other applications. In most cases, a lack of detailed yearly record keeping or unwillingness to spend the time required to collate data from multiple sources prevented farmer participants from providing the requested data, rather than data security concerns. It is possible that many producers do not maintain management records with the level of detail required for process-based modeling, or the effort required to collate management data from various sources puts an undue burden on producers. As stipulated by our IRB, reporting data was voluntary, thus whether unreported categories were due to lack of records, data privacy concerns, or the time/efforts required to collate records is unknown. In the absence of farmer-reported activity data, default management assumptions based on county- or regional-averages or agronomic recommendations can be used to specify model inputs. In some cases, use of default activity data such as planting or fertilizer application dates may not affect model outcomes, but missing management data for sensitive variables such as fertilizer application rates or manure amendments will result in greater uncertainty in model results.

According to previous studies, producers express concern about data security when there is a lack of trust between the agricultural community and the entity asking for the data, which may deter producers from participating in C crediting programs or research efforts [36]. To overcome this challenge, we recommend initiating collaborations between C accounting projects and data management software commonly used by producers, such as John Deere’s farm management software (APEX™). There is potential to align the data recorded automatically by advanced agricultural equipment software and the data needed to accurately and precisely model biogeochemical fluxes from cropland management. As precision agriculture and AI continue to expand, the viability of this option will increase. Further, the use of remote sensing tools to remotely monitor field activities and estimate soil properties using hyperspectral imagery can help overcome both soil measurement and management data challenges [59]. With these emerging technologies, data privacy and security to protect producer’s anonymity should be prioritized.

Furthermore, data that are timely and relevant to farmer’s management choices, such as changes in  profitability potential or fertilizer requirements, should be provided to farmer participants whenever possible. On-farm research has the potential to benefit all parties involved when the data production process becomes more collaborative and reciprocal. For example, farmer participants should be consulted in the study design phase to ensure the study is evaluating production systems most relevant to the region. Additionally, farmer participants should be provided with timely data reports describing the research findings relevant to their management decisions, including SOM and N stocks, water holding capacity, or profitability estimates. We propose the term “reciprocal research” to describe studies that are designed with the farmer participant’s interests in mind, rather than extracting information from agricultural communities without considering how the research can benefit the participants in the short-term. Reciprocal research, following principles of participatory action research common in the social sciences, has the potential to motivate system change by involving groups often excluded from the research process in the creation of new knowledge [16, 27].

## On-farm measurements can be assembled into a database to facilitate model improvement

**Fig. 1:**
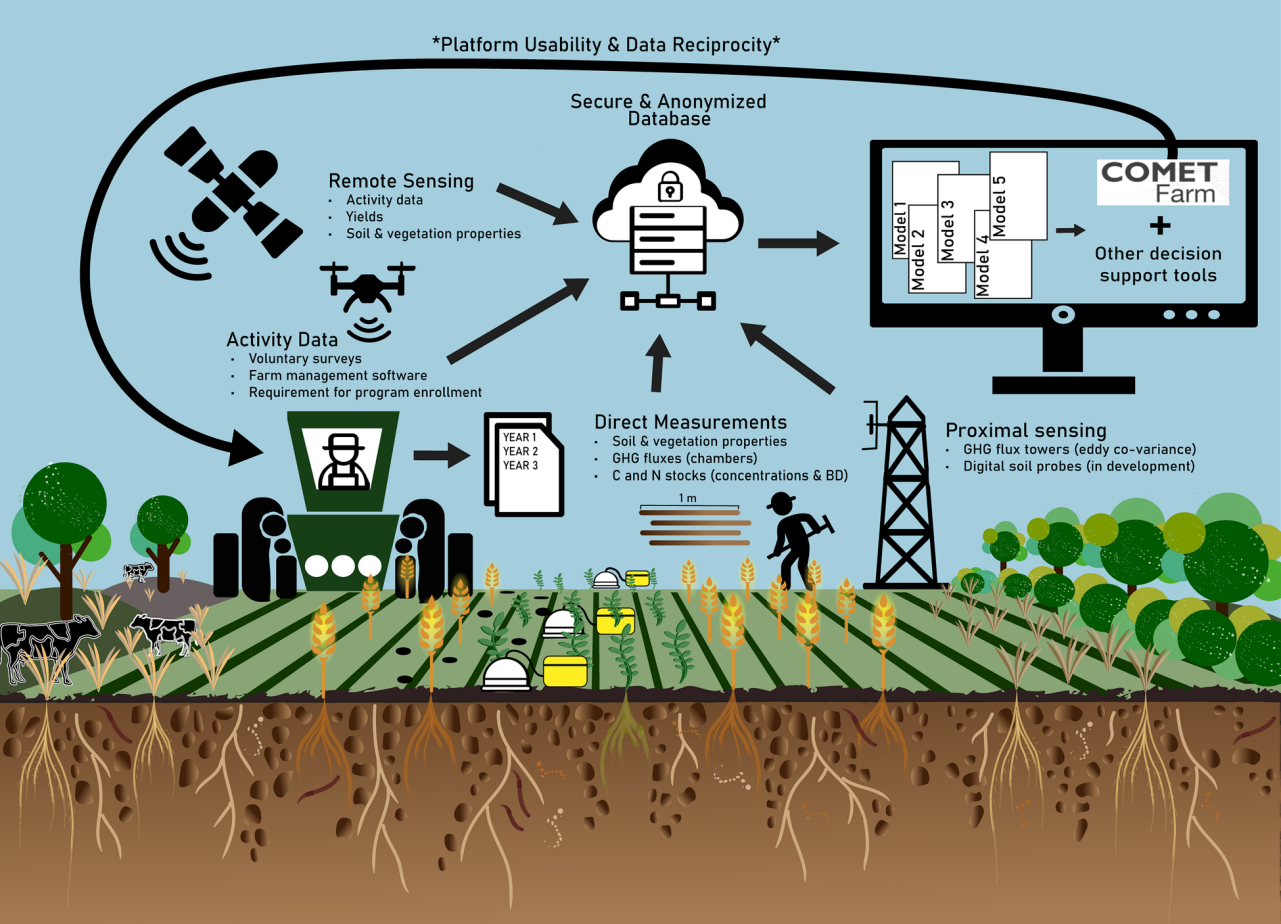
System framework for integrating on-farm measurements (directly measured, proximally sensed, and remotely sensed) into a secure and anonymized database that can be used for model improvements. Activity data is obtained from producers when readily available and remotely sensed whenever possible. Activity and measurement data are used simultaneously to improve existing model performance, conduct model intercomparisons, and build new models/ multi-model ensembles using artificial intelligence. Improved models can support decision support tools like COMET-Farm, which are expanded to include data of immediate relevancy to the producer following the principle of “reciprocal research.”

We propose a framework for integrating on-farm measurements and activity data into a secure, anonymized database to facilitate process-based model validation and improvements. As more on-farm studies are conducted, likely by private sector companies and research institutions, these data could be curated into an open-source database (Fig. 1). This database could facilitate model intercomparisons, multi-model ensembles, and systematic comparisons of LTE results to on-farm studies. Further, this database could provide a data source for model testing of decision support tools designed to easily quantify the benefits of climate-smart practice adoption, such as COMET-Farm and COMET-Planner. COMET-Farm was developed as a decision support tool that includes the DayCent process-based biogeochemical model, as well as a number of other greenhouse gas emission models and databases, to provide everyday users the ability to estimate agricultural GHG emissions and soil C sequestration through a web-based tool that does not require any modeling or other technical expertise [42]. Like most process-based models, DayCent and the associated COMET tools were developed mostly using data from LTEs. With improved sources of on-farm data, process-based models and decision support tools could be improved to better serve the end users and ensure the platform represents the range of agricultural management systems used across the country.

A key element of the proposed data platform should be providing the information needed to incentivize and facilitate the adoption of climate-smart practices on as many acres as possible. This means ensuring a diversity of cropping systems are represented, the model results are easy to interpret, and information provided is relevant to the producer. As the diversity and complexity of agricultural practices evaluated using these simple tools increases, there will be continual need to parameterize new practices and validate model results against measured data. Further, models that integrate socio-economic variables and environmental outcomes are needed to assess the system-level outcomes of management change [67]. Following the idea of reciprocal research, practice outcomes that are more relevant to management decision making, such as profitability, resilience to weather extremes, or fertilizer requirements, should be provided to support adoption of new practices more effectively. Additionally, the burden of reporting data should be alleviated from the producer. Integrated data acquisition through farm data management software or remote sensing of field activities could be used to reduce the reporting and survey burden on producers [30]. Data privacy, security, and anonymization will continue to be challenges under this framework, as this is a top concern for many producers interested in participating in carbon markets and related programs [36]

## Conclusion

Improved management of cropland soils has the potential to be a significant climate mitigation and adaptation tool, one that private markets and federal programs are heavily investing in. Measuring and modeling changes in cropland SOC stocks and GHG emissions associated with adoption of climate-smart practices is a challenging, yet necessary, aspect of agriculture GHG accounting or carbon offset generation. To test, validate, and improve process-based models and decision support tools, more direct soil measurements from commercial farms can complement data from LTEs while providing management-relevant data to facilitate scaled adoption. Detailed management data (obtained from producers or remotely sensed) paired with direct field measurements can support model-readiness for the diverse management scenarios encountered on diverse agricultural lands, which is of particular importance as interest in modeling SOC stocks on commercial farms continues to proliferate. We suggest the creation of a secure and anonymized database of on-farm measurements and activity data to facilitate model improvements and our understanding of what drives the successful adoption of climate-smart practices.

## Data Availability

Due to the private nature of the management data obtained from farmer participants, the activity data discussed here are not publicly available.
